# Effectiveness of Humanized AI Avatars and Messenger Gender for Dental Postprocedure Instructions: Two Randomized Experiments

**DOI:** 10.2196/85621

**Published:** 2026-07-09

**Authors:** Dafna Benadof, Josefa Cabezas, Daniel Schwartz

**Affiliations:** 1 School of Dentistry, Universidad Andrés Bello, Echaurren 237, Santiago, 8370133, Chile, 56 227703157; 2 Department of Industrial Engineering, University of Chile, Santiago, Chile

**Keywords:** artificial intelligence, AI, dental care, randomized controlled trial, patient compliance, avatar

## Abstract

**Background:**

Postcare instructions play a vital role in preventive health care. Traditionally, information on post–dental procedure care is provided through printed leaflets or brief verbal explanations, which often fail to engage patients or ensure adherence. In contrast, emerging artificial intelligence (AI) systems designed to emulate human interaction represent a promising yet understudied approach to improving patient communication and understanding.

**Objective:**

This study evaluated the effectiveness of AI-generated avatars in communicating postprocedure dental instructions and determined how video characteristics, including avatar type (humanized, animated, or real person), AI identity disclosure, and avatar gender, impact intention to comply and comprehension.

**Methods:**

Two preregistered online randomized experiments were conducted. Experiment 1 (n=650) compared the effectiveness of a real person with humanized and animated AI avatars, with and without disclosure of the AI’s identity. Experiment 2 (n=256) tested the effects of avatar gender on patient intention to comply. The primary outcomes were self-reported likelihood of following instructions, returning to the dentist, and comprehension.

**Results:**

In experiment 1, humanized AI avatars were as effective as a real person in promoting intention to comply with instructions, while animated avatars significantly reduced this intention. AI disclosure did not affect intention to comply but reduced the reported likelihood of returning to the same dentist. In experiment 2, women reported a higher intention to comply with the instructions in the man avatar condition than in the woman avatar condition. Comprehension remained high and was unaffected across conditions.

**Conclusions:**

Humanized AI avatars are an effective tool for delivering postprocedure instructions. However, design choices such as visual realism and avatar gender significantly impact patient trust and intention to comply. Avatar design should balance efficacy and ethics.

## Introduction

Nonadherence to postoperative dental instructions is a major public health issue, as it directly affects recovery outcomes [1]. Such nonadherence can result in serious yet preventable complications such as peri-implantitis, infections, and implant failure [2,3], and it leads to substantial economic costs due to avoidable complications and additional treatments [4].

Effective communication is essential for improving adherence in preventive health care [5]. Traditional methods such as printed instructions are often ineffective, especially for individuals with limited health literacy, as they lack engagement and personalization [6-8]. In contrast, visual formats—such as videos and animated computer agents—enhance patient understanding and intention to comply [7,9]. These visual formats may also help to address time constraints faced by clinicians [7]. However, their limited personalization can reduce their effectiveness for culturally diverse or low-literacy populations [8].

Artificial intelligence (AI) offers emerging tools for scalable health communication. In this context, AI refers to AI-driven systems used for text generation, speech synthesis, and avatar-based delivery of standardized health instructions. These messages can be personalized based on patient characteristics, such as gender and communication style, with human-like avatars enhancing engagement [10-12]. A humanized AI avatar is designed to closely resemble a real human being in appearance and behavior. Anthropomorphism theory suggests that human-like agents elicit stronger social and emotional responses than less realistic representations [13], thereby enhancing message engagement and compliance. Evidence from a pilot study using a virtual health assistant to deliver colorectal cancer screening messages [14] suggests that patients are more likely to intend to complete screening when it is recommended by a race-concordant health care provider. Nonetheless, those effects remain preliminary and context-dependent, underscoring the need for further theory-driven and adequately powered evaluations.

Moreover, research suggests that gender stereotypes influence patient trust, with some evidence showing that women may trust providers who are men more due to implicit bias and role congruity theory [15,16]. In addition, several studies have demonstrated that the gender of avatars and embodied agents influences participant perceptions and behavior. For example, Siegel et al [16] showed that participants’ willingness to donate to a robot varied depending on the robot’s gendered voice and the participant’s own gender, with men donating more to a woman robot—an effect attributed to the activation of gender stereotypes. On the other hand, Baake et al [10] found that highly realistic avatars were perceived as more trustworthy than stylized ones across dimensions of expertise, integrity, and benevolence. Specifically, they found that man avatars were rated higher in expertise. Similarly, Eyssel and Hegel [12] manipulated the perceived gender of a robot through interchangeable visual features and found stereotype-consistent evaluations, whereby robots who are men were perceived as more agentic and suitable for technical tasks, while woman robots were perceived as more communal and suitable for caregiving roles.

Evidence from human-agent interaction research shows that similar gender stereotyping effects extend to embodied artificial agents (ie, avatars), influencing perceived credibility, authority, and user intention to comply even when task performance is identical [12,16]. These biases extend beyond AI contexts, affecting perceptions of competence and trustworthiness.

AI-driven personalization shows potential for improving patient engagement and adherence. It could also enhance autonomy and acceptability. However, ethical considerations remain relevant, as user preferences themselves may reflect internalized social stereotypes. Tailoring by identity risks reinforcing stereotypes or widening disparities if done incautiously [11,17]. Therefore, even preference-based personalization should be implemented cautiously and transparently to avoid reinforcing existing biases. Additionally, disclosing that a message is AI-generated can reduce perceived empathy and credibility, challenging the balance between human likeness and transparency [18]. Despite growing interest, real-world evidence on AI avatars in postcare communication is limited; most studies focus on attitudes toward AI tools rather than on patients’ responses or understanding [19]. Moreover, evidence is especially sparse regarding the role of disclosure or how avatar gender interacts with patient identity to affect adherence [10,11,18].

To address these gaps, 2 preregistered online randomized experiments were conducted to answer the following research questions in the context of delivering dental posttreatment instructions: (1) whether humanized and animated AI avatars can achieve comparable effects to a human speaker on information understanding and intention to comply and (2) whether avatar gender influences intention to comply and whether this effect varies by participant gender. The findings contribute to the growing literature on AI in health communication and inform the development of personalized, ethically responsible digital interventions for preventive care.

## Methods

### Experiment 1

The study, conducted in December 2023 as a preregistered randomized online experiment, investigated how humanized and nonhumanized AI-generated avatars impact intention to comply with postcare instructions in a health care setting (Penn Wharton Credibility Lab 154774). It specifically assessed whether AI-generated messages are as effective as those from a human and explored how disclosure of the message’s AI origin influences this effect.

Participants were told that they had to undergo dental implant surgery during a visit to the dentist, a procedure for which postoperative care is crucial. To encourage them to follow the instructions, they were told that the dentist features an instructional video on post–dental implant surgery care. All materials are available in Multimedia Appendix 1.

### Ethical Considerations

Ethics approval was obtained from the research ethics committee of the Faculty of Physical and Mathematical Sciences at the University of Chile (#019). All participants provided informed consent prior to participation by electronically approving a consent form. Data were collected and analyzed anonymously. Participants received compensation for their participation in the study (£0.75-£0.90; £1=US $1.27 as of December 2023). The person featured in the real-person video condition participated voluntarily and provided written informed consent authorizing the use of her image and likeness for research purposes. The experiments were preregistered at the Penn Wharton Credibility Lab (154774 and 164881). Although the Penn Wharton Credibility Lab is not a World Health Organization–accredited trial registry, the requirement for registration in a World Health Organization–accredited registry was waived because the study did not involve a direct medical intervention.

### Participants

The sample consisted of 650 adults (mean age 44.5, SD 14.82 years; n=329, 50.6% women). Participants were recruited through Prolific Academic (Prolific Academic Ltd), and a preintervention screening ensured that all resided in the United States and English was their first language. Table S1 in Multimedia Appendix 2 present summary statistics for the sample.

### Interventions

Participants were randomly assigned to 1 of 5 video conditions, all delivering identical dental posttreatment instructions. Videos and audio were generated using Synthesia (Synthesia Ltd), an AI-based platform that produces human-like digital avatars delivering scripted health information. The conditions varied by the type of spokesperson—a real person, a humanized AI avatar, or an animated AI avatar—and whether participants were informed that the video was AI generated. All videos featured a White speaker who was a woman. The five groups were as follows: (1) real person (baseline), (2) humanized AI, (3) animated AI, (4) disclosed humanized AI, and (5) disclosed animated AI. The humanized AI avatar was designed to closely approximate a real person’s appearance with near-photorealistic facial features and naturalistic expressions. The animated AI avatar used stylized, nonphotorealistic visual features. All videos delivered identical scripted content. Visual examples are provided in Multimedia Appendix 3 and Multimedia Appendix 4. Participants were not required to watch the entire video, but time spent on the video page was tracked and did not differ across conditions (>97% spent sufficient time to watch the complete video).

### Outcomes

The study’s primary outcomes were intention to comply and understanding. Intention to comply was assessed using 2 Likert-scale questions regarding the likelihood of following instructions and returning to the same dentist (ranging from 1 =“extremely unlikely” to 7 =“extremely likely”). Understanding was measured with a 5-item multiple-choice quiz based on the video content, developed with a dentist’s guidance and expertise. The study also tracked whether participants rewatched the video before answering, as they could normally do. Participants also answered additional questions about the video, including its clarity, usefulness, engagement, and the speaker’s voice and appearance. Each of the 4 AI avatar conditions was compared with the “real person” condition, which served as the baseline. Finally, participants provided their opinions on topics related to AI and health care and answered demographic and open-ended questions about comments on the videos and the survey.

### Statistical Analysis

As preregistered, we conducted linear regressions with robust SEs for the main outcome variables. Analyses were performed using Stata (version 18; StataCorp).

### Experiment 2

The first experiment examined whether a humanized AI video has the potential to deliver postcare instructions in health care. In the second randomized experiment, we examined the effect of patient-speaker gender congruence on intention to comply after the same dental procedure described in the first experiment. The study was preregistered (Penn Wharton Credibility Lab 164881), and ethics approval was obtained from the same institution as that for the first experiment.

### Participants and Experimental Conditions

All 256 participants (mean age 41.3, SD 13.71 years; n=128, 50% women) were residents of the United States and native English speakers recruited via the Prolific Academic panel. A total of 4 participants were excluded from an initial sample of 260 participants because they did not report their gender. As a robustness test, we conducted all main analyses using the gender classification from Prolific Academic (Tables S2 and S3 in Multimedia Appendix 5). We used preintervention screening to ensure that none of the participants had participated in the first experiment and to balance gender representation. Table S1 in Multimedia Appendix 2 shows the summary statistics for the sample.

Participants were randomly assigned to 1 of 2 experimental conditions in which they viewed a short video conveying the same content about postdental care as that used in the first experiment. The conditions varied depending on whether the speaker was a female-presenting or male-presenting humanized AI avatar. Both humanized avatars were White, appeared to be of a similar age, and were dressed in casual clothes (the woman avatar wore a blouse and the man avatar wore a jacket over a T-shirt). The key outcomes examined were participants’ intention to comply and understanding of the video. The measures and analyses used to assess these outcomes, including the questions and scales, were identical to those in experiment 1.

## Results

### Experiment 1 Results

#### Intention to Comply

**Figure 1.**
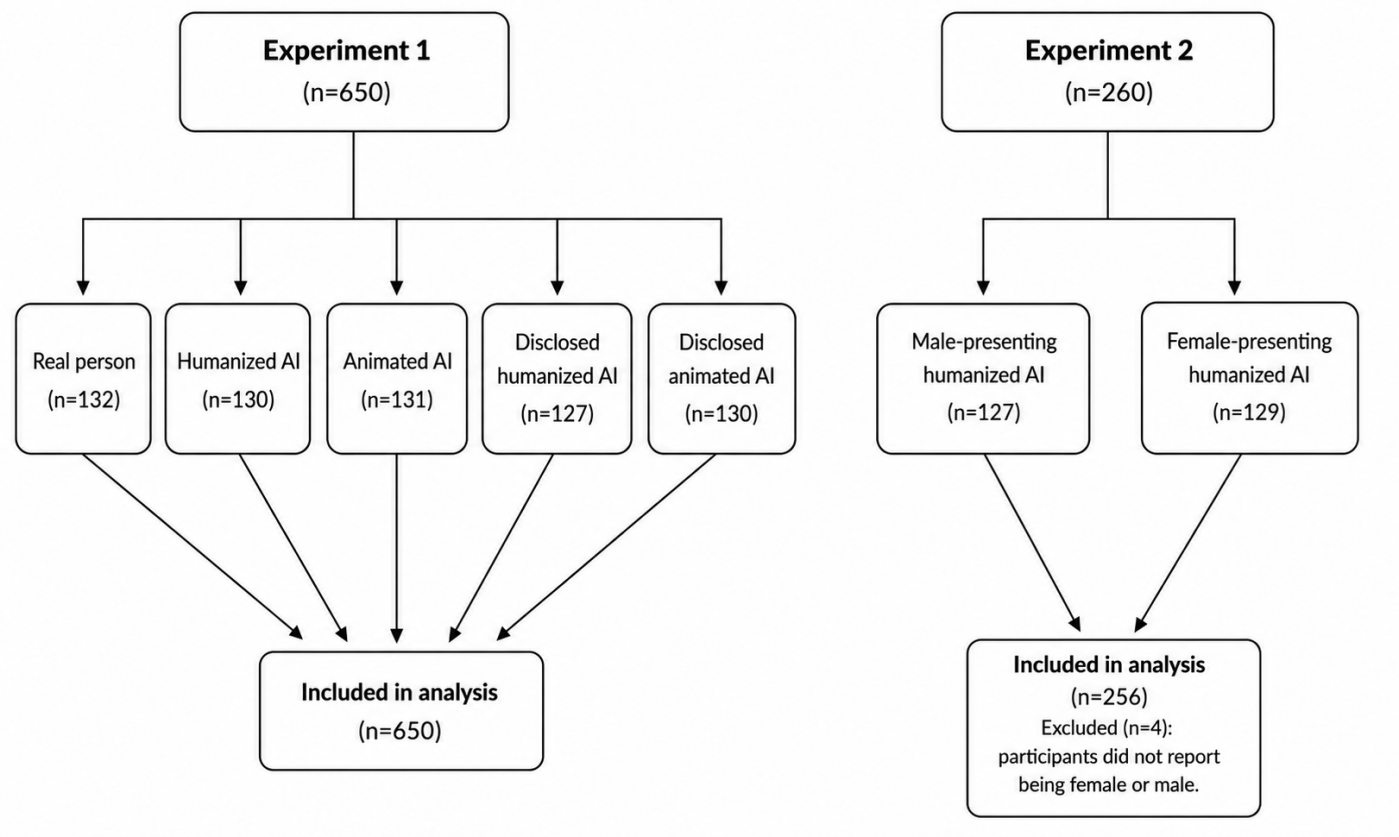
Participant flow and randomization for experiments 1 and 2.

Figure 1 summarizes the participant flow and randomization for both experiments. Participants were highly likely to follow postcare instructions when the video featured a real person (mean 6.52, SD 0.75) or a humanized AI avatar (mean 6.53, SD 0.89), with no significant difference between the 2, as indicated in Table 1 (95% CI −0.19 to 0.21; *P*=.94, which fell within the preregistered equivalence margin). However, those who viewed the animated AI avatar were significantly less likely to follow the instructions (mean 6.27, SD 1.05, 95% CI −0.48 to −0.04; *P*=.02; Cohen *d*=0.28). Disclosure effects on intention to comply were non-significant overall at the 95% confidence level. All results remained robust when using ordinal probit models (Tables S4 and S5 in Multimedia Appendix 6).

Consistently, participants who viewed a real person (mean 6.48, SD 0.71) or a humanized AI avatar (mean 6.32, SD 1.06) were similarly likely to continue with the same dentist, with no significant difference (95% CI −0.37 to 0.07; *P*=.17). However, those who watched the animated AI avatar were significantly less likely to do so (mean 5.97, SD 1.16, 95% CI −0.74 to −0.28; *P*<.01; Cohen *d*=0.53). Prior disclosure that the video was AI generated resulted in a lower willingness to return to the same dentist than the real-person condition (*P*<.01; Cohen *d*=0.35 and 0.37 for the disclosed humanized AI and disclosed animated AI conditions, respectively). Overall, humanized AI avatars performed comparably to real people, while animated AI avatars negatively affected patient intention to comply and follow-up intentions.

**Table 1.** Regression estimates for intention to comply and comprehension outcomes across video conditions (experiment 1; n=650)^a^.

Video conditions	Follow instructions	Return to dentist	Correct answers
Humanized AI^b^	0.008 (0.10)	−0.154 (0.11)	−0.097 (0.08)
Animated AI	−0.256^c^ (0.11)	−0.508^d^ (0.12)	−0.132 (0.09)
Disclosed humanized AI	−0.161 (0.10)	−0.312^e^(0.11)	−0.075 (0.08)
Disclosed animated AI	−0.100 (0.09)	−0.316^e^ (0.10)	−0.166 (0.09)
Constant (real person)	6.523^d^ (0.06)	6.477^d^ (0.06)	4.689^d^ (0.05)

a Values are regression coefficients (β) with robust SEs in parentheses.

b AI: artificial intelligence.

c *P*<.05.

d *P*<.001.

e *P*<.01.

#### Understanding

Participants who watched the real-person video had a high average understanding score (4.69 out of 5, SD 0.55), with 73% (97/132) answering all questions correctly. However, there were no significant differences in comprehension across any of the video conditions (*P*=.92 for the percentage achieving perfect scores and *P*≥.07 for the average score). Rewatching rates (146/650, 22.5% on average) also did not differ significantly between groups (*χ*²_4_=3.59; *P*=.46) and reflected real-world scenarios in which patients review instructions to ensure understanding. These results suggest that all videos were equally effective in conveying postcare instructions and that humanized AI videos can match real-person videos in supporting patient understanding and intention to comply.

#### Perceptions of the Video and Speaker

Ratings for the humanized AI condition were statistically indistinguishable from those for the real-person video on clarity, usefulness, and engagement (all *P*>.05). The animated AI condition scored lower on all 3 measures (all *P*<.01). With disclosure, animated AI clarity and usefulness scores were not lower than those of the real-person condition (*P*=.48 and *P*=.18, respectively), but engagement remained lower (*P*=.03). For the humanized AI condition, disclosure showed no clear effects on perceived clarity (*P*=.52) or perceived usefulness (*P*=.09) but showed a significant reduction in engagement (*P*<.01). Voice and pace ratings were similar for humanized AI and real-person conditions (*P*=.41), while appearance ratings were lower for both the humanized AI and animated AI conditions (both *P*<.01). Overall, disclosure modestly dampened evaluations of the humanized AI condition but improved appearance ratings for animated AI (disclosed animated AI vs disclosed humanized AI: *P*=.85). Multimedia Appendix 7 presents detailed results on participants’ perceptions of AI in health care. Tables S7 and S8 in Multimedia Appendix 8 report interactions with gender, showing that participants who are men and women responded very similarly when comparing the real person vs humanized AI conditions. Table S9 in Multimedia Appendix 9 presents interactions with education level, showing that the effectiveness of humanized AI was not moderated by education, while animated AI showed larger negative effects for participants with lower education. From the open-ended responses, participants referred to the person in the video as “the woman,” “the lady,” “character,” or “the presenter” rather than assigning her a professional role.

### Experiment 2 Results

#### Intention to Comply

On average, participants stated that they were “very likely” to follow the postprocedure care instructions (mean 6.36, SD 0.95), regardless of the avatar’s gender (*P*=.57). However, women reported a significantly lower intention to comply when the video featured a female-presenting humanized avatar (mean 6.37, SD 1.24) than when it featured the man version (mean 6.69, SD 0.50; 95% CI −0.66 to 0.00; *P*=.05; Cohen *d*=−0.35). In contrast, among men, there was no sizable difference in intention to comply between the female-presenting avatar (mean 6.28, SD 0.99) and the male-presenting avatar (mean 6.10, SD 1.07; 95% CI −0.17 to 0.55; *P*=.30; Cohen *d*=0.19). Consistently, Table 2 shows a significant interaction effect between participant gender and the avatar’s gender in the female-presenting video: compared to men, women reported a greater reduction of 0.52 Likert-scale points (95% CI −1.01 to −0.03; *P*=.04; Cohen *d*=−0.53). This interaction was driven by women responding more positively to man avatars than men did (*P*<.01) rather than responding negatively to woman avatars (*P*=.70).

Regarding the reported likelihood of returning to the same dentist, results show a similar pattern to that observed for the likelihood of following instructions, with women being less willing to return to the same dentist when the video featured a woman avatar than were men. However, the interaction effect was not statistically significant (95% CI −0.97 to 0.23; *P*=.23; Cohen *d*=−0.31).

**Table 2.** Regression estimates for intention to comply and comprehension outcomes by participant gender and artificial intelligence (AI) avatar gender (experiment 2; n=256)^a^.

Variables	Follow instructions	Return to dentist	Correct answers
Woman humanized AI	0.191 (0.18)	0.192 (0.22)	0.097 (0.18)
Woman	0.596^b^ (0.15)	0.500^c^ (0.21)	0.412^d^ (0.14)
Woman humanized AI × woman	−0.518^c^ (0.25)	−0.371 (0.31)	−0.119 (0.20)
Constant (man humanized AI)	6.097^b^ (0.14)	5.839^b^ (0.17)	4.403^b^ (0.13)

a Values are regression coefficients (β) with robust SEs in parentheses.

b *P*<.001.

c *P*<.05.

d *P*<.01.

#### Understanding

Across conditions, 76.6% (196/256) of participants answered all 5 questions correctly. In general, women had a significantly higher rate of correct answers (mean 4.80 out of 5, SD 0.48) than men (mean 4.45, SD 1.03; 95% CI 0.15-0.55; *P*<.01; Cohen *d*=0.44). Consistently, 83.6% (107/128) of women answered all 5 questions correctly, suggesting a high level of understanding of postcare instructions (89/128, 69.5% among men).

The gender of the speaker in the video had no significant impact on the number of correct answers for either men or women, with an average of 4.82 for the man humanized AI video and 4.79 for the woman humanized AI video (95% CI −0.19 to 0.15; *P*=.80; Cohen *d*=−0.04). This result did not vary across participant genders, as shown in Table 2 (95% CI −0.52 to 0.28; *P*=.56; Cohen *d*=−0.04). Consistently, there were no significant differences in the likelihood of rewatching the videos (65/256, 25.4% of participants did so, on average).

#### Video Characteristics

When examining whether there might be differences in the avatars to explain a woman’s better response to a man (vs woman) avatar video in intention to comply, we found that women evaluated both woman and man avatars very similarly, with no sizable differences, in clarity, usefulness, engagement, voice and pace, or appearance and confidence (all *P*>.05). Table S10 in Multimedia Appendix 10 provides detailed results for all variables.

Additionally, participants’ gender differences reveal that women’s higher level of intention to comply, regardless of the avatar’s gender, might be due to women (vs men) rating the information as clearer and being more willing to follow instructions, even when learning that the video was AI generated (both *P*<.05). None of these differences explained why women responded more positively to a male-presenting avatar than to a female-presenting avatar, but they may help explore targeted communications. As in experiment 1, participants commonly referred to the video presenter using gendered terms such as “man,” “woman,” “guy,” and “lady,” as well as pronouns (“he” and “she”).

## Discussion

### Principal Findings

This study evaluated AI-generated avatars for postoperative dental instructions. In experiment 1, the high level of realism of the humanized AI avatar was intentional, aiming to test whether near-human realism could achieve effects comparable to those of a real person. Results show that humanized AI matched a real speaker on intention to comply and comprehension, whereas animated avatars reduced intention to comply despite similar comprehension, suggesting that visual realism—not content—drives the observed responses. Regarding clinical significance, the small to medium effect size for the animated AI condition is comparable to effect sizes that have predicted real-world health behaviors in prior research [20]. Moreover, AI disclosure had mixed effects: it modestly lowered usefulness and engagement for humanized avatars but improved clarity and usefulness, although not engagement, for animated avatars. Transparency thus interacts with visual realism.

In experiment 2, women responded more positively to man avatars, while men did not exhibit a significant gender preference. This interaction highlights the complex dynamics between speaker characteristics and participant identity, indicating both opportunities and challenges for personalization strategies in AI-based health communication. Importantly, this interaction occurred without any difference in comprehension, suggesting that using a man avatar may enhance adherence among women without negatively affecting understanding for any participants.

Our finding that humanized AI avatars achieved functional equivalence with real speakers in terms of compliance intentions is consistent with the findings of Nasser Oesterreich et al [21], who found that photo-realistic avatars matched real photographs on trustworthiness ratings, and with those of Krieger et al [14], whose pilot study showed that virtual health assistants increased screening intentions. Our results suggest that this perceptual similarity extends to behavioral outcomes. Similarly, pilot studies by Haider et al [19] and Suárez et al [22] found that realistic AI avatars improved satisfaction and trust [19,22]; our study shows that comparable benefits persist in patient-facing instruction, although we measured intention to comply rather than attitudes toward the AI avatars. In addition, the compliance penalty we observed for animated avatars echoes the findings of Baake et al [10], who found that less realistic agents reduced trustworthiness in health communication. By holding informational content constant while varying the visual presentation (animated, humanized, or real person), our findings further demonstrate that these visual variations, rather than content clarity, drive compliance intentions.

Our findings on gender interactions align with broader patterns in avatar perception research. Eyssel and Hegel [12] found stereotype-driven preferences favoring man avatars on competence dimensions. We observed a similar pattern among women in a medical instruction context, while men showed no preference, suggesting that gender effects may be asymmetric in health care settings. Our disclosure findings show reduced engagement following AI revelation, parallel to prior observations following chatbot disclosure [18].

As AI tools become increasingly integrated into health care, it is crucial to examine how they might unintentionally reinforce social biases or stereotypes. Our findings show that women responded more favorably to man avatars. This may reflect internalized gender norms or stereotypes, potentially shaped early in life, that create expectations about professional roles and persist into adulthood [23]. This pattern is consistent with well-known social psychology theories, such as role congruity theory [15], which explains how stereotypes about ability can continue to exist.

Therefore, although personalization offers potential benefits, it must be ethically grounded. Designing avatars to match user preferences—whether in gender, race, or demeanor—should be done cautiously to avoid reinforcing stereotypes or limiting patient autonomy. Alternative approaches, such as user-driven selection of humanized AI avatars to deliver information, may enhance perceived autonomy; however, whether such approaches increase intentions to comply merits future investigation. These considerations point to the importance of using a population health framework to develop inclusive digital health interventions that enhance equity rather than exacerbate disparities.

This study contributes to the expanding literature on digital health communication and AI by empirically validating prior theoretical claims. Our results align with findings that human likeness and anthropomorphic cues increase trust and engagement [10,19]. They also offer nuanced insights into how AI disclosure can moderate user responses [24].

Furthermore, our findings support the view that comprehension of medical instructions can remain high even in AI-mediated formats, consistent with research on the role of animated computer agents in health literacy [7]. However, few studies have evaluated how these tools impact long-term adherence or real-world behaviors, which remains a critical avenue for future research.

### Limitations

The study was conducted in a single clinical context—post–dental implant care—and included a sample restricted to English-speaking participants in the United States, who identified as man or woman, which may limit the generalizability of the findings, particularly regarding gender interactions among nonbinary or gender-nonconforming individuals. Moreover, the observed effects could also reflect authority bias arising from attire cues (eg, a jacket), which might be confounded with gender effects, or from perceived professional role, as participants likely interpreted the speaker as a health care professional rather than as the dentists themselves who performed the procedure. Therefore, broader evaluations across multiple clinical settings and more diverse populations, including populations with substantially lower health literacy, are needed to assess the scalability and inclusiveness of AI-based communication tools. Additional measurement instruments may also be needed to detect improvements beyond the real-person baseline, which may have been subject to ceiling effects, as a benchmark to be reached. Additionally, experiment 2’s lack of a men human control group prevents assessment of whether women’s stronger preference for men avatars stems from an AI-specific positive bias or from more general health care gender biases, consistent with role congruity theory [15].

Future research should also examine how individual preferences, cultural background, and implicit biases influence user responses to AI avatars. Longitudinal studies that track actual health behaviors and outcomes will be essential to determine whether the observed effects on intention to comply translate into real-world adherence and improved health outcomes.

The findings suggest that humanized AI avatars could supplement or even replace human communicators in certain postcare contexts without compromising message effectiveness. Health care systems may leverage these tools to reduce clinician workload, provide repeatable instructions, and support patients with lower health literacy [7,25]. Designing avatars with clear voices, friendly tones, and culturally sensitive features—while strategically deciding when and how to disclose their AI nature—can maximize their impact [11,18,25]. If implemented ethically and equitably, these tools could help reduce postprocedure complications and lower health care costs, especially in resource-limited settings.

### Conclusions

This study demonstrates that humanized AI avatars can be as effective as human providers in delivering postprocedure instructions, provided that their visual design and disclosure are thoughtfully managed. These findings contribute to the growing body of literature on AI in health communication and highlight the potential for personalized, scalable, and ethical digital care strategies. Moving forward, it is essential to ensure that such technologies are inclusive, empirically validated, and tailored to support equity in health care delivery.

## Supplementary material

10.2196/85621 Multimedia Appendix 1 Experimental material.

10.2196/85621 Multimedia Appendix 2 Summary statistics.

10.2196/85621 Multimedia Appendix 3 Video stimuli for each condition in experiment 1.

10.2196/85621 Multimedia Appendix 4 Video AI avatars for each condition in experiment 2.

10.2196/85621 Multimedia Appendix 5 Robustness checks with other female variables for experiment 2.

10.2196/85621 Multimedia Appendix 6 Robustness checks with ordinal probit.

10.2196/85621 Multimedia Appendix 7 Perceptions on video content and the speaker, and about AI healthcare (Experiment 1).

10.2196/85621 Multimedia Appendix 8 Regression estimates with gender interactions (Experiment 1).

10.2196/85621 Multimedia Appendix 9 Regression estimates with education level interactions (Experiment 1).

10.2196/85621 Multimedia Appendix 10 Regression estimates for video evaluation and communicator perception outcomes (Experiment 2).

10.2196/85621 Checklist 1 CONSORT-eHealth (version 1.6.1) submission or publication form.

## Abbreviations

AI: artificial intelligence
