# Supplementary material for: Effectiveness of Humanized AI Avatars and Messenger Gender for Dental Postprocedure Instructions: Two Randomized Experiments
Source: JMIR AI. 2026 Jul 9;5:e85621. doi: 10.2196/85621 (PMC13349325; doi:10.2196/85621)
Supplement: Multimedia Appendix 1 [file ai-v5-e85621-s001.docx]

### **Multimedia Appendix 1: Experimental material**

Please consider the following situation:

You're in the dentist's office, dealing with a relentless toothache. Your dentist breaks the news that your tooth has decayed extensively and is beyond saving. The most effective solution is to replace the decayed tooth with a dental implant.

The dentist extracts the damaged tooth and inserts a titanium implant into your jawbone. In the next few months, a new artificial tooth will be attached to this implant, and before you know it, you'll have a natural-looking tooth. With proper care, this dental implant promises to serve you well for many years ahead.

To ensure you know how to take care of your new implant, your dentist provides you with a video [generated through artificial intelligence^^[[1]](#footnote-1)^^] detailing the post-procedure instructions.

Please click next to watch the video.

[Next page]

Please pay close attention to this post-procedure video [generated through artificial intelligence], which contains vital information about the care you should take after your dental surgery.

[video]

You've just undergone a significant dental procedure, and taking the right care of your implant is crucial for its success.

Firstly, manage any discomfort with over-the-counter pain relievers. If those aren't sufficient, inform us to prescribe something stronger.

Follow the course of the prescribed antibiotics to prevent infection. This is important even if you're feeling better partway through the course.

Oral hygiene is vital. Continue brushing and flossing your other teeth as usual but avoid the surgical area for the first 24 hours. Then, you can gently clean the area using a soft toothbrush, being careful to avoid causing discomfort or bleeding.

In terms of diet, stick to soft foods such as soups, pasta, and yogurts for the first three days to avoid putting unnecessary pressure on the implant. As your mouth heals and you become more comfortable, you can gradually reintroduce harder foods.

Make sure to attend your follow-up appointment, scheduled two weeks after surgery, to confirm everything is healing as expected.

Finally, should you have any questions or concerns, or if you experience significant pain or swelling that doesn't decrease after a few days, please contact us immediately.

Thank you for your attention.

[end video]

Feel free to pause and rewatch the video as needed. Once you're ready, please click next to answer some questions about the video.

Please answer the following questions after watching the video:

(1: Extremely unlikely; 2: Very unlikely; 3: Somewhat unlikely; 4: Neither likely nor unlikely; 5: Somewhat likely; 6: Very likely; 7: Extremely likely)

- How likely are you to follow the post-care instructions as provided in the video?
- Based on the video, how likely are you to continue with the same dentist for a check-up?
- How likely are you to watch the video again if you forgot the post-care instructions?
- How likely are you to recommend this video to someone who has had a dental implant procedure?

Please answer these general questions about the video:

- How clear did you find the instructions in the video? (1: Not clear at all; 2: Slightly clear; 3: Somewhat clear; 4: Moderately clear; 5: Quite clear; 6: Very clear; 7: Extremely clear)
- How would you rate the information provided in the video? (1: Not useful at all; 2: Slightly useful; 3: Somewhat useful; 4: Moderately useful; 5: Quite useful; 6: Very useful; 7: Extremely useful)
- How engaging did you find the video content? (1: Not engaging at all; 2: Slightly engaging; 3: Somewhat engaging; 4: Moderately engaging; 5: Quite engaging; 6: Very engaging; 7: Extremely engaging)

Indicate your level of agreement with the following statements:

(1: Strongly disagree; 2: Disagree; 3: Somewhat disagree; 4: Neither agree nor disagree; 5: Somewhat agree; 6: Agree; 7: Strongly agree)

- The speaker’s tone of voice and pace of speech in the video made the information easy to understand.
- The speaker’s appearance and confidence made me trust the information provided.

Based on the video, please answer the following questions as best as you can. If you want, you can watch the video again at the bottom of this page. [order of alternatives was random]

- If over-the-counter pain relievers aren't sufficient to manage your discomfort after the dental implant procedure, what should you do next?

a) Increase the dosage of the over-the-counter pain relievers

b) Purchase a different brand of over-the-counter pain relievers

c) Contact the dentist for a stronger prescription

d) Rinse your mouth with cold water until the pain is alleviated

e) Endure the pain as it's normal and part of the healing process

- If you're feeling better partway through the course of antibiotics, what should you do?

a) Stop taking the antibiotics

b) Reduce the dosage of antibiotics

c) Continue the full prescription as directed

d) Contact the dentist

e) Save the remaining antibiotics for future use

- After the dental implant surgery, when are you advised to start cleaning the surgical area, and what kind of toothbrush should you use?

a) Immediately after the surgery with a soft toothbrush

b) One week after the surgery with a soft toothbrush

c) One week after the surgery with a hard toothbrush

d) The day after the surgery with a soft toothbrush

e) The day after the surgery with a hard toothbrush

- What type of diet is recommended for the first few days after the surgery?

a) Only hard foods

b) Only soft foods

c) Any type of food is okay

d) Avoid eating altogether

e) Only liquids

- Which of the following symptoms necessitates immediate contact with your dentist after the procedure?

a) Mild discomfort

b) The taste of metal in your mouth

c) Light swelling

d) Significant pain or swelling that doesn't decrease after a few days

e) The sensation that the implant is not in the right place

Please answer these questions about your perception of the video

- If you found out that the presenter in the video was actually a bot created by artificial intelligence, would that change your responses to the first questions about the video? (1: Extremely unlikely; 2: Very unlikely; 3: Somewhat unlikely; 4: Neither likely nor unlikely; 5: Somewhat likely; 6: Very likely; 7: Extremely likely)
- How comfortable are you with the idea of artificial intelligence (AI) being involved in your healthcare? (1: Extremely uncomfortable; 2: Very uncomfortable; 3: Somewhat uncomfortable; 4: Neither comfortable nor uncomfortable; 5: Somewhat comfortable; 6: Very comfortable; 7: Extremely comfortable)
- How would you categorize the presenter in the video? (1: Definitely human; 2: Probably human; 3: Very probably human; 4: Uncertain; 5: Probably AI bot; 6: Very probably AI bot; 7: Definitely AI bot)

Please answer these final questions about you

- What is your age?: [open text box]
- What is your gender?
  - Female
  - Male
  - Non-binary
  - Prefer not to say
- What is the highest level of education?
  - Some high school, incomplete
  - High school or equivalent
  - Some college, incomplete
  - Undergraduate college degree (BS, BA)
  - Graduate degree (MA, PhD, MBA, etc)
- Which race or ethnicity best describes you? [non-mandatory]
  - White / Caucasian
  - Black or African American
  - Hispanic or Latino
  - Asian
  - Native American or Alaska Native
  - Native Hawaiian or other Pacific Islander
  - Other: [text]

Please provide any comments about the video (content and format). [Text]

Were the instructions difficult to follow? Please let us know any comments about the survey. [Text]

1. For the AI disclosed conditions. [↑](#footnote-ref-1)
