# Supplementary material for: Effectiveness of Humanized AI Avatars and Messenger Gender for Dental Postprocedure Instructions: Two Randomized Experiments
Source: JMIR AI. 2026 Jul 9;5:e85621. doi: 10.2196/85621 (PMC13349325; doi:10.2196/85621)
Supplement: Multimedia Appendix 2 [file ai-v5-e85621-s002.docx]

### **Multimedia Appendix 2: Summary statistics**

Table S1. Summary statistics of the samples

| Variable | Experiment 1 (N=650) | Experiment 2 (N=256) |
| --- | --- | --- |
| Mean Age (SD) | 44.51 (14.82) | 41.29 (13.71) |
| Female | 50.62% | 50.00% |
| Education |  |  |
| Graduate degree | 16.46% | 14.06% |
| High school or equivalent | 16.77% | 11.72% |
| Some college, incomplete | 26.00% | 31.64% |
| Some high school, incomplete | 0.92% | 2.34% |
| Undergraduate degree (BS, BA) | 39.85% | 40.23% |
| Race/Ethnicity |  |  |
| Asian | 5.85% | 10.16% |
| Black or African American | 15.85% | 20.31% |
| Hispanic or Latino | 4.31% | 6.64% |
| Native American or Alaska Native | 0.62% | 0.78% |
| Native Hawaiian or Pacific Islander | 0.46% | 0.39% |
| Other | 2.62% | 1.95% |
| White / Caucasian | 70.31% | 59.77% |
