# Supplementary material for: Effectiveness of Humanized AI Avatars and Messenger Gender for Dental Postprocedure Instructions: Two Randomized Experiments
Source: JMIR AI. 2026 Jul 9;5:e85621. doi: 10.2196/85621 (PMC13349325; doi:10.2196/85621)
Supplement: Multimedia Appendix 3 [file ai-v5-e85621-s003.docx]

### **Multimedia Appendix 3: Video stimuli for each condition in experiment 1**

**
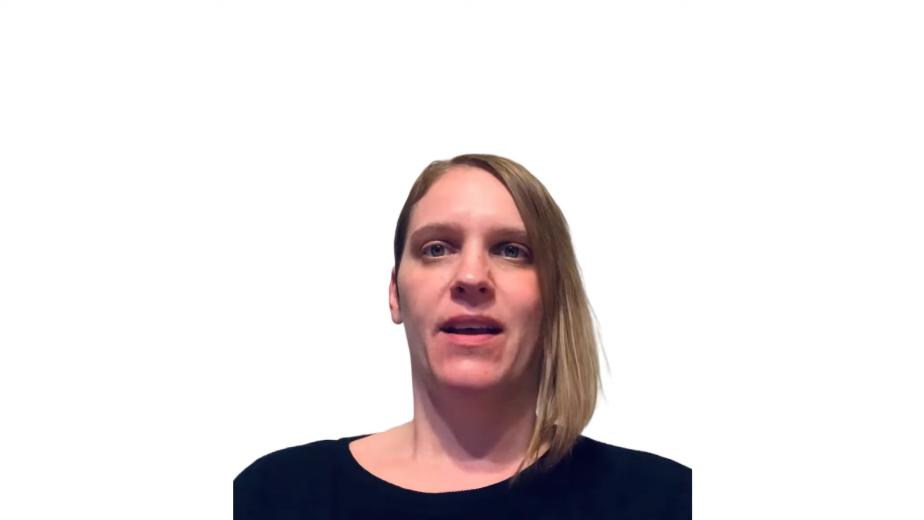
**

Figure 1: Real person


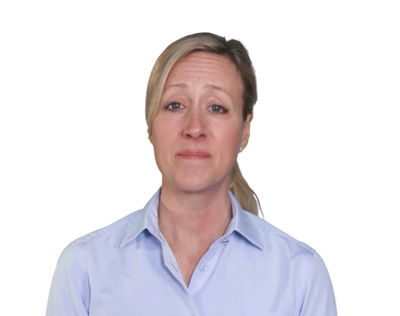


Figure 2: Humanized AI-generated avatar


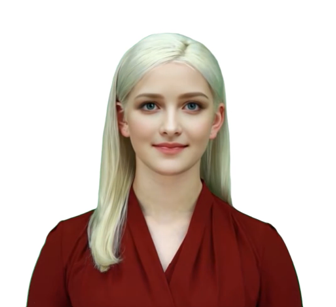


Figure 3: Animated AI-generated avatar
