# Supplementary material for: Effectiveness of Humanized AI Avatars and Messenger Gender for Dental Postprocedure Instructions: Two Randomized Experiments
Source: JMIR AI. 2026 Jul 9;5:e85621. doi: 10.2196/85621 (PMC13349325; doi:10.2196/85621)
Supplement: Multimedia Appendix 4 [file ai-v5-e85621-s004.docx]

### **Multimedia Appendix 4: Video AI avatars for each condition in experiment 2**


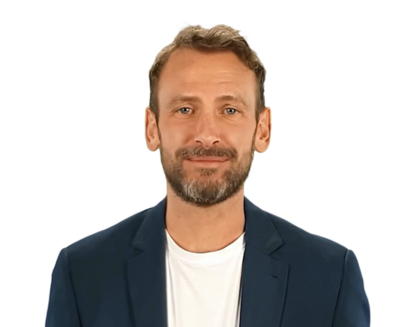


Figure 4: Male humanized AI-generated avatar


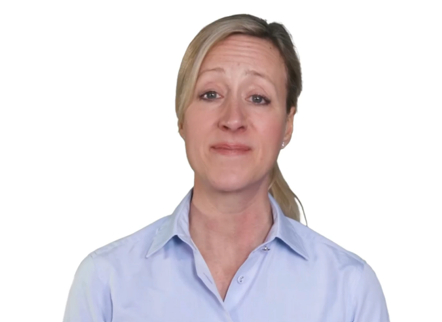


Figure 5: Female humanized AI-generated avatar
