# Supplementary material for: Effectiveness of Humanized AI Avatars and Messenger Gender for Dental Postprocedure Instructions: Two Randomized Experiments
Source: JMIR AI. 2026 Jul 9;5:e85621. doi: 10.2196/85621 (PMC13349325; doi:10.2196/85621)
Supplement: Multimedia Appendix 5 [file ai-v5-e85621-s005.docx]

### **Multimedia Appendix 5: Robustness checks with other female variables for experiment 2**

Table S2: Regressions for intention to comply and understanding with the Female variable that addresses four missing gender values with prolific demographic information for those participants (N = 260).

|  | (1)  Follow instructions | (2)  Return to dentist | (3)  Correct answers |
| --- | --- | --- | --- |
| Female Humanized AI | 0.179  (0.18) | 0.202  (0.22) | 0.078  (0.18) |
| Female (Corrected) | 0.562^***^  (0.15) | 0.515^*^  (0.21) | 0.384^**^  (0.14) |
| Female Humanized AI × Female (Corrected) | -0.485^*^  (0.25) | -0.387  (0.30) | -0.090  (0.20) |
| Constant (Male Humanized AI) | 6.109^***^  (0.13) | 5.828^***^  (0.17) | 4.422^***^  (0.13) |
| Robust standard errors in parentheses.* *P* < .05, ** *P* < .01, *** *P* < .001 | | | |

Table S3: Regressions for intention to comply and understanding with the Female variable that considers Prolific Academic’s demographic information for all participants (N = 260).

|  | (1)  Follow instructions | (2)  Return to dentist | (3)  Correct answers |
| --- | --- | --- | --- |
| Female Humanized AI | 0.172  (0.18) | 0.158  (0.22) | 0.047  (0.18) |
| Female (Prolific info) | 0.551^***^  (0.15) | 0.452^*^  (0.21) | 0.304^*^  (0.14) |
| Female Humanized AI × Female (Prolific info) | -0.463  (0.25) | -0.289  (0.30) | -0.022  (0.20) |
| Constant (Male Humanized AI) | 6.111^***^  (0.13) | 5.857^***^  (0.17) | 4.460^***^  (0.12) |
| Robust standard errors in parentheses.* *P* < .05, ** *P* < .01, *** *P* < .001  For four participants, self-reported gender differed from their Prolific profile (potentially outdated). The interaction in model (1) yields *P* = .06. | | | |
