# Supplementary material for: Effectiveness of Humanized AI Avatars and Messenger Gender for Dental Postprocedure Instructions: Two Randomized Experiments
Source: JMIR AI. 2026 Jul 9;5:e85621. doi: 10.2196/85621 (PMC13349325; doi:10.2196/85621)
Supplement: Multimedia Appendix 6 [file ai-v5-e85621-s006.docx]

### **Multimedia Appendix 6: Robustness checks with ordinal probit**

Table S4. Ordered probit regression (Experiment 1)

|  | (1)  Follow instructions | (2)  Return to dentist |
| --- | --- | --- |
| Humanized AI | 0.109 | -0.108 |
|  | (0.15) | (0.15) |
| Animated AI | -0.299* | -0.539*** |
|  | (0.14) | (0.14) |
| Disclosed Humanized AI | -0.240 | -0.366** |
|  | (0.14) | (0.13) |
| Disclosed Animated AI | -0.142 | -0.380** |
|  | (0.14) | (0.13) |
| Log likelihood | -648.984 | -768.762 |
| N | 650 | 650 |

Robust standard errors in parentheses. * *P* < .05, ** *P* < .01, *** *P* < .001

Table S5. Ordered probit regression (Experiment 2)

|  | (1)  Follow instructions | (2)  Return to dentist |
| --- | --- | --- |
| Female Humanized AI | 0.267 | 0.168 |
|  | (0.19) | (0.18) |
| Female Participant | 0.857*** | 0.555** |
|  | (0.20) | (0.19) |
| Female Humanized AI × Female Participant | -0.631*  (0.29) | -0.326  (0.28) |
|  |  |  |
| Log pseudolikelihood | -254.317 | -324.445 |
| N | 256 | 256 |

Robust standard errors in parentheses. * *P* < .05, ** *P* < .01, *** *P* < .001
