# Supplementary material for: Effectiveness of Humanized AI Avatars and Messenger Gender for Dental Postprocedure Instructions: Two Randomized Experiments
Source: JMIR AI. 2026 Jul 9;5:e85621. doi: 10.2196/85621 (PMC13349325; doi:10.2196/85621)
Supplement: Multimedia Appendix 7 [file ai-v5-e85621-s007.docx]

### **Multimedia Appendix 7: Perceptions on video content and the speaker, and about AI healthcare (Experiment 1)**

Participants evaluated the Humanized AI similarly to the real-person video. Participants who watched the real person rated the videos as having high clarity and usefulness (M = 6.36 and M = 6.14, respectively) and reported moderate engagement (M = 4.68). None of these ratings were significantly different from those given by participants who watched the Humanized AI (M = 6.49 for clarity, M = 6.15 for usefulness, and M = 4.36 for engagement; all *P* > .05). Conversely, participants who viewed the Animated AI video rated it lower across all perception measures, including clarity (M = 6.05), usefulness (M = 5.78), and engagement (M = 3.89), all *P* < .01.

Interestingly, when participants were informed beforehand that the video was AI-generated, evaluations of the clarity and usefulness of the Animated AI video were not reduced compared to the real person (M = 6.29; *P* = .48 and M = 5.98; *P* = .18, respectively). Engagement continued to be much lower than it was with no disclosure (M = 4.27; *p* = 0.03). For the Humanized AI, clarity remained relatively high and not significantly different from the responses for the real-person video (M = 6.30; *P* = .52), but usefulness was marginally reduced (M = 5.91; *P* = .09) as well as engagement (M = 4.04; *P* < .01). The results with disclosure might suggest that people have a lower expectation of AI-generated information.

For questions directly related to the speakers, participants rated the real-person video highly regarding the speaker's tone of voice and pace (M = 6.06) and appearance (M = 5.73). The Humanized AI video received a similar rating for voice (M = 5.95; *P* = .41) but a lower score for appearance (M = 5.12; *P* < .01), indicating that it closely approximated the real-person experience despite some limitations in appearance. The Animated AI video, however, was rated significantly lower in both voice (M = 5.60) and appearance (M = 4.60; all *P* < .01), which may explain the reduced compliance and engagement in this condition. Furthermore, the Disclosed Humanized AI video saw a slight reduction in the tone of voice ratings (M = 5.76; *P =* .03) and a drop in appearance (M = 4.93; *P* < .01), while the Disclosed Animated AI video had a voice score of M = 5.86 (*P =* .15, compared to the real-person video) and an appearance rating of M = 4.89 (*P <* .01). These findings suggest that revealing the AI’s nature diminished overall perceptions of the speaker.

Overall, most participants do not express (conscious) discomfort when asked about the idea of artificial intelligence (AI) being involved in their healthcare (24.5% reported some degree of discomfort, 16.7% neither comfortable nor uncomfortable, and 58.8% somewhat, very, or extremely comfortable). Consistently, when participants were asked how likely they would be to follow the post-care instructions if they discovered the presenter was AI-generated, 89.2% reported being either somewhat, very, or extremely likely. Neither result showed differences across conditions, highlighting the difference between revealed and declared preferences methods.

Table S6. Regression estimates for video evaluation and communicator perception outcomes across video conditions (main effects only; Experiment 1; N = 650).

|  | (1) Instruction Clarity | (2) Information Usefulness | (3) Engagement | (4) Voice & Pace | (5) Appearance |
| --- | --- | --- | --- | --- | --- |
| Humanized AI | 0.129 (0.093) | 0.010 (0.116) | -0.320 (0.199) | -0.107 (0.130) | -0.612*** (0.166) |
| Animated AI | -0.318** (0.113) | -0.365** (0.122) | -0.796*** (0.186) | -0.458*** (0.138) | -1.132*** (0.159) |
| Disclosed Humanized AI | -0.064 (0.101) | -0.231 (0.134) | -0.642*** (0.193) | -0.297* (0.136) | -0.806*** (0.165) |
| Disclosed Animated AI | -0.071 (0.100) | -0.159 (0.118) | -0.413* (0.191) | -0.199 (0.136) | -0.843*** (0.158) |
| Constant (Real Person) | 6.364*** (0.064) | 6.144*** (0.074) | 4.682*** (0.119) | 6.061*** (0.080) | 5.735*** (0.088) |

Robust standard errors in parentheses. * *P* < .05, ** *P* < .01, *** *P* < .001
