# Supplementary material for: Effectiveness of Humanized AI Avatars and Messenger Gender for Dental Postprocedure Instructions: Two Randomized Experiments
Source: JMIR AI. 2026 Jul 9;5:e85621. doi: 10.2196/85621 (PMC13349325; doi:10.2196/85621)
Supplement: Multimedia Appendix 8 [file ai-v5-e85621-s008.docx]

### **Multimedia Appendix 8: Regression estimates with gender interactions (Experiment 1)**

Table S7. Regression estimates for intention to comply and understanding across video conditions (Experiment 1; N = 650).

|  | (1) Follow instructions | (2) Return to dentist | (3) Correct answers |
| --- | --- | --- | --- |
| Humanized AI | 0.004 (0.16) | -0.089 (0.17) | -0.188 (0.14) |
| Animated AI | -0.406* (0.19) | -0.536** (0.18) | -0.130 (0.14) |
| Disclosed Humanized AI | -0.156 (0.15) | -0.202 (0.16) | -0.165 (0.15) |
| Disclosed Animated AI | -0.115 (0.15) | -0.125 (0.14) | -0.033 (0.14) |
| Female | 0.245 (0.13) | 0.332** (0.12) | 0.260** (0.09) |
| Humanized AI × Female | -0.017 (0.20) | -0.156 (0.22) | 0.145 (0.16) |
| Animated AI × Female | 0.320 (0.21) | 0.063 (0.23) | -0.001 (0.17) |
| Disclosed Humanized AI × Female | -0.032 (0.20) | -0.240 (0.22) | 0.146 (0.16) |
| Disclosed Animated AI × Female | 0.009 (0.19) | -0.393 (0.21) | -0.278 (0.18) |
| Constant | 6.406*** (0.11) | 6.319*** (0.10) | 4.565*** (0.08) |

Robust standard errors in parentheses. * *P* < .05, ** *P* < .01, *** *P* < .001

Table S8. Regression estimates for video evaluation and communicator perception outcomes across video conditions (Experiment 1; N = 650).

|  | (1) Instruction Clarity | (2) Information Usefulness | (3) Engagement | (4) Voice & Pace | (5) Appearance |
| --- | --- | --- | --- | --- | --- |
| Humanized AI | 0.056 (0.14) | -0.027 (0.18) | -0.199 (0.28) | -0.062 (0.18) | -0.701** (0.23) |
| Animated AI | -0.464** (0.16) | -0.406* (0.18) | -0.652** (0.25) | -0.522** (0.20) | -1.058*** (0.22) |
| Disclosed Humanized AI | -0.038 (0.14) | -0.110 (0.19) | -0.409 (0.28) | -0.462* (0.22) | -0.683** (0.24) |
| Disclosed Animated AI | -0.095 (0.15) | -0.173 (0.19) | -0.060 (0.28) | -0.174 (0.21) | -0.686** (0.23) |
| Female | 0.124 (0.13) | 0.210 (0.15) | 0.153 (0.24) | 0.066 (0.16) | -0.100 (0.18) |
| Humanized AI × Female | 0.124 (0.19) | 0.048 (0.23) | -0.244 (0.40) | -0.091 (0.26) | 0.178 (0.33) |
| Animated AI × Female | 0.309 (0.22) | 0.087 (0.24) | -0.303 (0.38) | 0.136 (0.27) | -0.157 (0.32) |
| Disclosed Humanized AI × Female | -0.063 (0.20) | -0.248 (0.27) | -0.458 (0.39) | 0.307 (0.27) | -0.224 (0.33) |
| Disclosed Animated AI × Female | 0.034 (0.20) | 0.007 (0.24) | -0.687 (0.38) | -0.053 (0.28) | -0.291 (0.32) |
| Constant | 6.304*** (0.09) | 6.043*** (0.11) | 4.609*** (0.17) | 6.029*** (0.12) | 5.783*** (0.12) |

Robust standard errors in parentheses. * *P* < .05, ** *P* < .01, *** *P* < .001
