# Supplementary material for: Effectiveness of Humanized AI Avatars and Messenger Gender for Dental Postprocedure Instructions: Two Randomized Experiments
Source: JMIR AI. 2026 Jul 9;5:e85621. doi: 10.2196/85621 (PMC13349325; doi:10.2196/85621)
Supplement: Multimedia Appendix 9 [file ai-v5-e85621-s009.docx]

### **Multimedia Appendix 9: Regression estimates with education level interactions (Experiment 1)**

Table S9. Regression estimates for intention to comply and understanding by education level (ordinal education scale; interactions with video condition; Experiment 1; N = 650).

|  | (1) Follow Instructions | (2) Return to Dentist | (3) Correct Answers |
| --- | --- | --- | --- |
| Humanized AI | 0.197 (0.279) | -0.549 (0.347) | 0.016 (0.303) |
| Animated AI | -0.899* (0.353) | -1.557*** (0.416) | 0.084 (0.291) |
| Disclosed Humanized AI | -0.326 (0.321) | -0.380 (0.340) | 0.028 (0.279) |
| Disclosed Animated AI | -0.220 (0.356) | -0.411 (0.391) | -0.843* (0.407) |
| Education (ordinal) | -0.031 (0.050) | -0.116* (0.052) | -0.005 (0.054) |
| Humanized AI × Education | -0.054 (0.081) | 0.111 (0.098) | -0.032 (0.082) |
| Animated AI × Education | 0.188 (0.100) | 0.303** (0.114) | -0.064 (0.083) |
| Disclosed Humanized AI × Education | 0.047 (0.083) | 0.018 (0.090) | -0.029 (0.078) |
| Disclosed Animated AI × Education | 0.034 (0.094) | 0.029 (0.106) | 0.184 (0.101) |
| Constant | 6.633*** (0.178) | 6.891*** (0.183) | 4.708*** (0.207) |

Robust standard errors in parentheses. * *P* < .05, ** *P* < .01, *** *P* < .001

Education is coded on a five-point ordinal scale (1 = some high school incomplete, 5 = graduate degree).
