# Supplementary material for: Effectiveness of Humanized AI Avatars and Messenger Gender for Dental Postprocedure Instructions: Two Randomized Experiments
Source: JMIR AI. 2026 Jul 9;5:e85621. doi: 10.2196/85621 (PMC13349325; doi:10.2196/85621)
Supplement: Multimedia Appendix 10 [file ai-v5-e85621-s010.docx]

### **Multimedia Appendix 10: Regression estimates for video evaluation and communicator perception outcomes (Experiment 2)**

Table S10. Regression estimates for video evaluation and communicator perception outcomes (Experiment 2; N = 256).

|  | (1) Instruction Clarity | (2) Information Usefulness | (3) Engagement | (4) Voice & Pace | (5) Appearance |
| --- | --- | --- | --- | --- | --- |
| Female Humanized AI | 0.059 (0.187) | 0.263 (0.201) | -0.114 (0.299) | 0.044 (0.224) | -0.065 (0.292) |
| Female Participant | 0.337 (0.171) | 0.462* (0.208) | 0.056 (0.271) | 0.499** (0.191) | 0.162 (0.286) |
| Female Humanized AI × Female Participant | -0.158 (0.243) | -0.196 (0.273) | -0.245 (0.423) | -0.092 (0.287) | 0.207 (0.393) |
| Constant | 6.032*** (0.134) | 5.661*** (0.149) | 4.129*** (0.193) | 5.516*** (0.159) | 4.823*** (0.214) |

Robust standard errors in parentheses. * *P* < .05, ** *P* < .01, *** *P* < .001
